# ColBuilder: flexible structure generation of crosslinked collagen fibrils

**DOI:** 10.1093/bioinformatics/btaf278

**Published:** 2025-05-05

**Authors:** Debora Monego, Matthias Brosz, Johanna Buck, Vsevolod Viliuga, Paulius Greicius, Jaewoon Jung, Torsten Stuehn, Matthias Schmies, Yuji Sugita, Frauke Gräter

**Affiliations:** Max Planck Institute for Polymer Research, Mainz 55128, Germany; Heidelberg Institute for Theoretical Studies, Heidelberg 69117, Germany; Heidelberg Institute for Theoretical Studies, Heidelberg 69117, Germany; Institute for Scientific Computing, Heidelberg University, Heidelberg 69120, Germany; Max Planck Institute for Polymer Research, Mainz 55128, Germany; Heidelberg Institute for Theoretical Studies, Heidelberg 69117, Germany; Institute for Scientific Computing, Heidelberg University, Heidelberg 69120, Germany; Max Planck Institute for Polymer Research, Mainz 55128, Germany; Science for Life Laboratory, Solna 172 21, Sweden; Department of Biochemistry and Biophysics, Stockholm University, Stockholm 114 18, Sweden; Max Planck Institute for Polymer Research, Mainz 55128, Germany; Max Planck School Matter to Life, Heidelberg 69120, Germany; Computational Biophysics Research Team, RIKEN Center for Computational Science, Kobe 650-0047, Japan; Theoretical Molecular Science Laboratory, RIKEN Pioneering Research Institute, Wako 351-0198, Japan; Max Planck Institute for Polymer Research, Mainz 55128, Germany; Max Planck Institute for Polymer Research, Mainz 55128, Germany; Computational Biophysics Research Team, RIKEN Center for Computational Science, Kobe 650-0047, Japan; Theoretical Molecular Science Laboratory, RIKEN Pioneering Research Institute, Wako 351-0198, Japan; Max Planck Institute for Polymer Research, Mainz 55128, Germany; Heidelberg Institute for Theoretical Studies, Heidelberg 69117, Germany; Institute for Scientific Computing, Heidelberg University, Heidelberg 69120, Germany

## Abstract

**Motivation:**

Collagen fibrils are fundamental building blocks of connective tissues, yet generating accurate molecular models of their structure remains challenging due to their hierarchical organization and complex crosslinking patterns.

**Results:**

ColBuilder has been developed to automate the generation of atomistic models of crosslinked collagen fibrils and facilitate the setup of molecular simulations. The tool integrates homology modeling, higher order structure generation and optimization to build complete fibril structures with precise control over sequence composition, crosslinking patterns, and dimensions. Users can explore different collagen sequences, manipulate crosslink chemistry through mixed ratios and densities, and generate fibrils of varying diameter and length. All-atom molecular dynamics simulations of 335nm-long fibrils validate the generated structures, showing excellent agreement with experimental measurements of D-band periodicity and force-extension behavior.

**Availability and implementation:**

ColBuilder is available both as an open-source command-line application and through a web interface at https://colbuilder.mpip-mainz.mpg.de.

## 1 Introduction

Collagen fibrils are integral components of the extracellular matrix, playing a crucial role in the structural integrity and mechanical properties of various tissues. Understanding the structural and functional dynamics of collagen fibrils is essential for advancing tissue engineering strategies and elucidating mechanisms of collagen-related diseases. However, this understanding remains challenging due to the hierarchical complexity and intricate assembly processes of collagen fibrils (Kadler *et al.* 1996, Shoulders and Raines 2009). While experimental techniques such as X-ray crystallography (Orgel *et al.* 2006) and nuclear magnetic resonance (Jelinski *et al.* 1980, Peixoto *et al.* 2013) have provided valuable atomic-level information, they often struggle to resolve the full complexity of collagen structures, particularly in capturing heterogeneity and flexibility.

The structural complexity of collagen fibrils is further complicated by tissue-specific variations in crosslinking patterns (Eyre and Wu 2005, Avery *et al.* 2009). These crosslinks, formed between lysine and hydroxylysine residues, evolve throughout tissue development and aging, with alterations in their density and composition being hallmarks of various pathological conditions (Snedeker and Gautieri 2014). The specific arrangement of these crosslinks within the characteristic D-banded structure of collagen fibrils—consisting of gap and overlap regions—creates unique mechanical behaviors that are difficult to capture experimentally (Depalle *et al.* 2015). Slight modifications in crosslinking chemistry can lead to dramatic changes in tissue mechanics (Willett *et al.* 2010, Kwansa *et al.* 2016), making molecular-level understanding of these structure–function relationships crucial for developing targeted interventions in collagen-related diseases and designing biomimetic materials with controlled mechanical properties.

Computational modeling and molecular dynamics (MD) simulations have emerged as powerful complementary tools to address these experimental limitations. These approaches enable detailed exploration of collagen fibril conformations (Monti *et al.* 2005, Streeter and de Leeuw 2010, Gautieri *et al.* 2011), dynamics (Zapp *et al.* 2020), and interactions (Perumal *et al.* 2008, Streeter and de Leeuw 2011), as well as their crosslinking patterns (Gautieri *et al.* 2011). Early studies using molecular modeling (Uzel and Buehler 2011) revealed, for instance, that crosslinks particularly affect the C-terminal domain of collagen fibrils, where they prevent intermolecular sliding and significantly alter the distribution of mechanical strain throughout the structure. Despite these valuable insights, several fundamental challenges persist in the computational modeling of collagen: the size disparity between atomic-scale interactions and tissue-level properties creates difficulties for multiscale modeling; the diversity of collagen types across species and tissues necessitates sequence-specific approaches; and the accurate representation of posttranslational modifications remains difficult. Among these challenges, perhaps one of the most immediate practical hurdles is that preparing accurate initial models for such simulations can be labor-intensive and error-prone, often requiring the integration of multiple tools, data sources, and specialized expertise. While dedicated tools exist for generating models of specific biological systems, such as CHARMM-GUI and Packmol for membranes (Martínez *et al.* 2009, Lee *et al.* 2019) and nanodiscs (Qi *et al.* 2019), there has been a notable absence of analogous tools for filamentous proteins like collagen.

To address this gap, some of us previously developed ColBuilder, a database offering pre-compiled atomistic models of the D-band region of the Collagen I fibril (Obarska-Kosinska *et al.* 2021). We now introduce the tool ColBuilder, which significantly expands upon its predecessor’s capabilities by enabling end-to-end generation of complex collagen fibrils. ColBuilder implements a template-based modeling strategy, using experimentally derived collagen structures as a foundation for generating diverse fibril models. Key advancements include: (i) homology modeling capabilities for collagens from different species and compositions, (ii) advanced crosslinking manipulation, allowing users to mix different crosslinks at varying ratios or control crosslinks density, and (iii) full control over the final fibril’s diameter and length. These features directly address the limitations of current computational approaches by providing a streamlined, accurate, and flexible tool for collagen fibril modeling.

ColBuilder is provided as both an open-source command line application built with Python (https://github.com/graeter-group/colbuilder) and an accompanying web interface (https://colbuilder.mpip-mainz.mpg.de). To facilitate the use of our models in MD simulations, we also provide a topology generation option and Amber force field parameters for different lysine-derived crosslink types. We have conducted a comprehensive validation study using computational and experimental benchmarks to assess the accuracy and reliability of ColBuilder’s output. The versatility of ColBuilder extends its value beyond MD simulations, with potential applications in cryo-electron microscopy model fitting and as templates for protein design. Finally, we hope that the approach we used in ColBuilder will be transferable to model other filamentous protein materials, such as fibronectin and intermediate filaments.

## 2 Materials and methods

**Figure 1.**
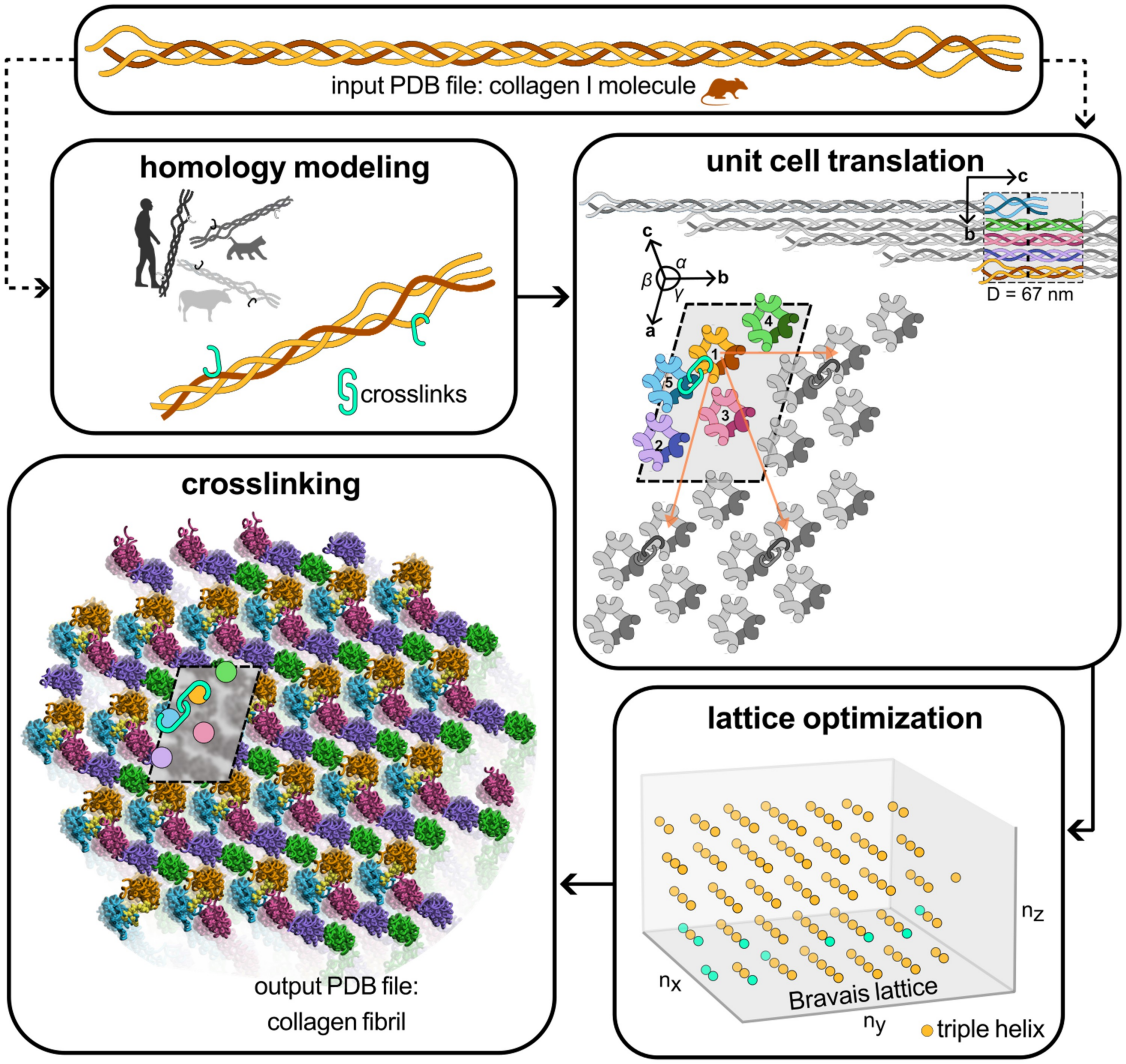
Workflow diagram of ColBuilder, showing the main steps including choice of input structure, generation of copies and their translation to form the higher order structure, lattice optimization in Bravais lattice, and crosslinking to form the final optimized collagen fibril model.

Figure 1 illustrates the workflow of ColBuilder for collagen fibril generation. The primary input is a 3D structure of a single collagen triple helix in PDB format, including unit cell symmetry information. ColBuilder offers two main pathways: (i) direct fibril generation, where users specify desired dimensions and crosslinking parameters and (ii) sequence modification and modeling, where users can input custom crosslinking specifications or alter the template’s amino acid sequence. In both cases, the resulting higher order structure is optimized within a Bravais lattice, with appropriate crosslinking of triple helices.

### 2.1 Homology modeling and initial structure generation

ColBuilder accommodates two distinct input pathways, each initiating a different processing workflow. Users can either use a previously optimized model of Collagen I in PDB format and containing collagen fibril’s unit cell definition, or input a custom collagen amino acid sequence in the FASTA format. In the case of direct structure input, ColBuilder utilizes the provided structure without the need for homology modeling. However, when an amino acid sequence is provided, the software employs a homology modeling process to generate the initial structure.

For amino acid sequence inputs, our homology modeling process uses a template structure of rat collagen Type I, derived from the model used in the previous version of ColBuilder (Obarska-Kosinska *et al.* 2021). The process begins with a two-step alignment procedure implemented using Biopython (Cock *et al.* 2009) to preserve the structural integrity of the collagen triple helix. First, we align the three chains within the template collagen molecule, ensuring the conservation of the characteristic Gly–X–Y repetitive pattern; this aligned triple helix template is then aligned with the target sequence using MUSCLE v3.8.31 (Edgar 2004). This method allows for the introduction of species-specific variations or desired mutations while maintaining the core collagen structure.

MODELLER performs the homology modeling using a refinement protocol that prioritizes backbone stability. This approach substitutes amino acids without significantly altering the overall backbone conformation, which is critical for maintaining the triple helical structure characteristic of collagen. We validated this procedure for 18 different species with a range of sequence identities (≈70%−98%) to the rat Collagen I template. Structural consistency was assessed by per-residue RMSD calculations and preservation of key collagen motifs, particularly the Gly–X–Y pattern. For all sequences tested, the per-residue RMSD was consistently below 2 Å and the average axial rise per triplet (i.e. the distance between consecutive Gly–C${}^{\alpha }$ in the triple helix) is in agreement with the expected value of approximately 8.6 Å for collagen triple helices (Bella *et al.* 1994) (see Supplementary Fig. S1 and Supplementary Table S1), demonstrating the robustness of our method.

During ColBuilder’s development, we evaluated AlphaFold (Jumper *et al.* 2021) as a potential alternative for generating initial collagen structures. We conducted systematic testing with AlphaFold 3 (Abramson *et al.* 2024) using collagen sequences from three species with varying evolutionary distances (for details, see Supplementary Table S2 and Supplementary Fig. S2). Our analysis showed that while individual collagen regions can be predicted with moderate accuracy, the interfaces where crosslinks form between multiple helices remain particularly problematic. Specifically, AlphaFold struggles to position lysine residues in appropriate proximity for crosslink formation—a critical requirement for accurate collagen fibril modeling. These findings likely stem from several challenges specific to collagen: its repetitive Gly–X–Y pattern differs from the diverse sequences these models were trained on and challenges multiple sequence alignments, its ∼1000 amino acid chains exceed optimal prediction lengths, and its quaternary structure involves unique interchain stabilizing interactions, including some between strongly posttranslationally modified residues. These limitations support our decision to implement a template-based homology modeling approach for ColBuilder’s initial structure generation.

ColBuilder’s homology modeling feature greatly enhances its versatility compared to its predecessor, enabling users to generate and analyze collagen structures from different species or with specific mutations. While our approach produces accurate molecular-level structures, we advise users to carefully examine models generated from sequences that deviate significantly from our template (rat collagen, Type I). Additionally, it is important to note that fibril-level organization may vary between species, as experimental structural data at this scale is primarily derived from native rat Type I collagen (Orgel *et al.* 2006).

### 2.2 Higher order structure generation

Building upon the initial collagen template, ColBuilder generates higher order structures using UCSF Chimera v1.15’s crystal contacts command (Pettersen *et al.* 2004). The process begins with a collagen molecule coordinate file in PDB format, defining atoms $A=a_{1},\ldots ,a_{N}$ at positions $Q=\left(q_{1},\ldots ,q_{N}\right)$, along with a user-specified contact distance. The algorithm extracts crystallographic information from the input file, including lattice parameters (a,b,c,α,β,γ), space group ($G_{SP}$), and crystal orientation matrix C. This information defines the unit cell and guides the generation of the higher order fibril structure. ColBuilder then generates multiple symmetry copies of the unit cell using Euclidean transformation matrices T=(R,t), where R and t represent rotation and translation, respectively. Specifically, each atom in the new set $A^{\prime }$ undergoes a transformation according to the equation:


(1) $$T(q_{i})=Rq_{i}+t,$$

where i=1,…,N. The new symmetry copies have positions $Q^{\prime }=T(q_{1},\ldots ,q_{N})=\left(q_{1}^{\prime },\ldots ,q_{N}^{\prime }\right)$ (bennet2010understanding).

The contact distance parameter (*dc*) in UCSF Chimera’s crystal contacts tool determines the cutoff distance for including symmetry-related copies of the unit cell. As this distance increases, more symmetry operations are applied, incorporating unit cells that are further from the origin in the crystal lattice. This results in the generation of larger fibril structures. The exact relationship between contact distance and fibril diameter is complex, depending on the unit cell parameters, space group, and specific symmetry operations of the collagen crystal structure. Supplementary Figure S3 shows the empirical relationship between the contact distance parameter and the resulting fibril radius generated by ColBuilder, demonstrating how users can control the size of the generated microfibril structure by adjusting this parameter. We note that for contact distances dc≤15, only up to a couple of complete unit cells are included, and increasing *dc* has no effect in the overall fibril diameter.

A critical step in the higher order structure generation process is the identification and removal of steric clashes between atoms from different symmetry copies. Our algorithm ensures physically realistic structures by considering the van der Waals radii ($r_{w,i},r_{w,j}$) of interacting atoms. It generates a coordinate file of the higher order structure and transformation matrices for each symmetry copy, enabling subsequent refinement on a discrete Bravais lattice. The resulting system is an ensemble of models $\mu =(\mu_{1},\ldots ,\mu_{M})$, where each model $\mu_{m}$ contains information about its crosslinks $A_{m}^{c}\subset A_{m}$ (if present), Euclidean transformation $T_{m}$, and Bravais lattice point $p_{m}$. Any crosslink present in the model is characterized by type (divalent or trivalent; for details, see next section), residue ID, residue name, chain ID, and atomic positions $Q_{m}^{c}=(q_{m,i}^{c},\ldots ,q_{m,k}^{c})$ in Cartesian coordinates.

#### 2.2.1 Structure optimization

To efficiently represent the quasicrystalline nature of collagen fibrils, we map higher order structures from Cartesian space $\left(R^{3}\right)$ to a discrete lattice $\left((n_{x},n_{y},n_{z})\in Z^{3}\right)$ using the crystal orientation matrix C [Equation (2)]. Transformations between spaces are performed using $p=C^{-1}t$, where t represents the Cartesian translation vector and p the corresponding lattice point.


(2) $$\begin{array}{c} C=\left(\begin{array}{ccc} a & b\cdot \;\text{cos}\;\gamma & c\cdot \;\text{cos}\;\beta \\ 0 & b\cdot \;\text{sin}\;\gamma & c\cdot \left(\text{cos}\;\alpha -\frac{\;\text{cos}\;\beta \cdot \;\text{cos}\;\gamma }{\;\text{sin}\;\gamma }\right)\\ 0 & 0 & \sqrt{c^{2}-c_{xz}^{2}-c_{yz}^{2}} \end{array}\right). \end{array}$$

Initial structural analysis of the microfibril revealed a centrosymmetrical pattern with an inversion center at the lattice origin, consistent with the triclinic unit cell of collagen. However, we observed a 35% higher point density within 10 nm of the origin compared to regions beyond 50 nm, indicating denser packing of collagen triple helices near the center. This is a consequence of the finite size of the fibril, resulting in less triple helices at the boundaries of the fibril. Connectivity analysis showed that 73% of molecules were crosslinked at one end, 18% at both ends, and 9% remained unconnected, highlighting the need for structural optimization.

**Figure 2.**
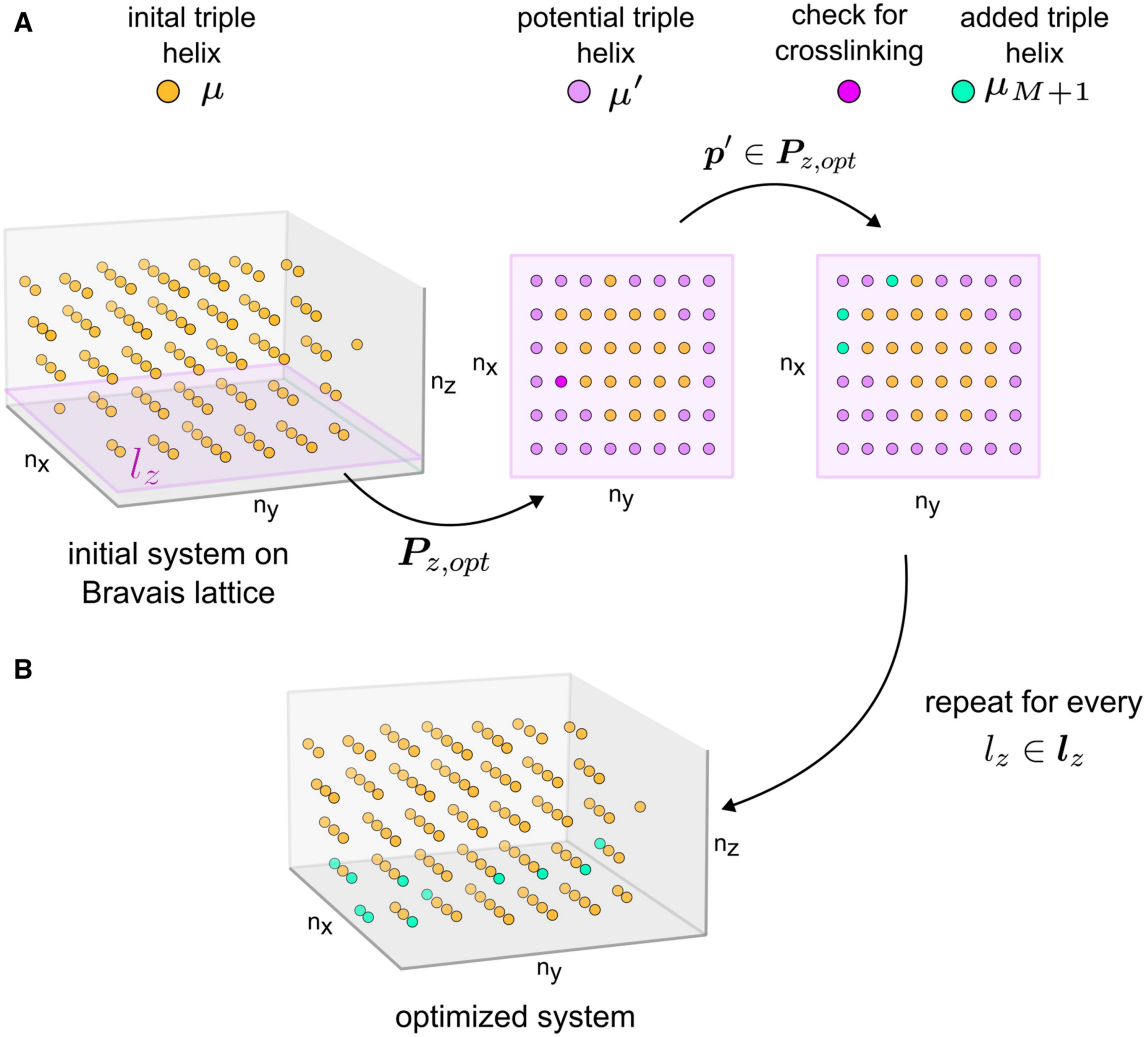
Structural optimization process of a collagen fibril on a Bravais lattice. Starting with an initial system of triple helices μ arranged on a Bravais lattice (A), the process selects a layer $l_{z}$ for optimization. This layer spans an optimization space $P_{m}$ containing potential new triple helices $\mu^{\prime }$. Each potential triple helix $\mu^{\prime }$ is evaluated for possible crosslinking with existing helices in the model. Triple helices that can be crosslinked are then added to the original system, resulting in an optimized structure (B). This procedure can be repeated for any number of $n_{x},n_{y}$-layers, gradually enhancing the fibril’s structural integrity while maintaining its lattice arrangement.

To address these issues, we developed a layer-by-layer optimization approach on the Bravais lattice. The process begins by defining the solution space using $\delta =(\delta_{x},\delta_{y},\delta_{z})$, which restricts possible solutions to a finite set (Fig. 2A). The $\delta_{z}$ parameter selects the number of $n_{x},n_{y}$-layers for optimization. For instance, setting $\delta_{z}=2$ defines the solution space as the two upper ($n_{z,\text{min}}+\delta_{z},n_{z,\text{min}}+\delta_{z}-1$) and two lower ($n_{z,\text{max}}-\delta_{z},n_{z,\text{max}}-\delta_{z}+1$) layers from $l_{z}$ for optimization. For each layer $l_{z}\in l_{z}$, we define a set of points $P_{z}=(p_{i,x},p_{i,y},l_{z})$ with $i=1,\ldots ,N_{z}$. We then determine the extreme values in the $n_{x},n_{y}$ dimensions to create vectors $l_{x}$ and $l_{y}$, which span a rectangular plane $P_{z,\text{rec}}=(l_{x},l_{y},l_{z})$ (Fig. 2A). The optimization space is defined as $P_{z,\text{opt}}=P_{z,\text{rec}}\setminus P_{z}$.

The algorithm proceeds by randomly selecting points $p^{\prime }\in P_{z,\text{opt}}$ and mapping them to Cartesian space to obtain transformation matrices $T^{\prime }$ and translation vectors $t^{\prime }$. We translate the model $\mu_{1}$ crosslink positions by $t^{\prime }$ and check intercrosslink distances between the new potential model $\mu^{\prime }$ and existing models μ (Fig. 2A). Contact criteria are defined based on a 0.3 nm cutoff for interatomic distances, which is typical for noncovalent interactions in proteins. If these criteria are met between $\mu^{\prime }$ and existing models, we incorporate $\mu^{\prime }$ into the system as $\mu_{M+1}$; otherwise, we discard it. The solution space is updated after each iteration, and the process continues until the current layer’s solution space is exhausted. We then move to the next layer and repeat the process until all layers are optimized (Fig. 2B).

The optimized models and transformation matrices are combined using UCSF Chimera’s *matrixset* command to generate an atomistic structure of the collagen microfibril. Postprocessing involves cutting each triple helix to the user-specified length (up to 335 nm) and capping termini with neutral acetyl or N-methyl groups, preparing the structure for subsequent MD simulations.

Our optimization approach resulted in substantial enhancements to the collagen microfibril’s structural properties. We observed a 28% improvement in packing uniformity, quantified by the reduction in point density standard deviation across the lattice. The optimized structures showed excellent agreement with experimental data. We measured an average D-band periodicity of 66.95±0.01 nm, closely aligning with the experimentally inferred value of 67 nm. To calculate this periodicity, we applied *K*-means clustering (Macqueen 1967, Pedregosa *et al.* 2011) (*k* = 10) to the *z*-coordinates of crosslinking residues, identifying distinct bands along the fibril axis. The D-band was then computed as the sum of the gap and overlap distances between adjacent clusters, with distances below 38 nm classified as overlaps and those above as gaps. Additionally, we observed an average lateral molecular spacing of 1.44±0.12 nm, which falls within the range of reported experimental values (1.1 nm for completely dry tissue to 1.8 nm for fresh tissue; Fratzl *et al.* 1993). This lateral spacing was calculated by projecting the molecule coordinates onto a plane perpendicular to the fibril’s principal axis, followed by a nearest-neighbor analysis in each quadrant around individual molecules to determine local spacings.

The flexibility of our approach allows for the exploration of different packing configurations by modifying the solution space. For example, considering up to the fourth layer in the $n_{z}$ dimension ($\delta_{z}=4$) results in a Bravais lattice with a slightly different geometrical arrangement of points, particularly for layers closer to the lattice origin. This adaptability enables fine-tuning of the optimization process to meet specific structural requirements or investigate various packing configurations.

Notably, our Bravais lattice-based optimization method demonstrated a 10-fold increase in computational efficiency for basic operations compared to traditional Cartesian approaches. In conclusion, our procedure provides an efficient and flexible approach to generate realistic collagen microfibril structures. The resulting models not only exhibit improved structural properties but also align closely with experimental observations, making them valuable for further computational studies of collagen mechanics and function.

### 2.3 Crosslink specification for collagen microfibrils

Collagen microfibrils are stabilized by intermolecular covalent crosslinks derived from lysine and hydroxylysine residues in the telopeptide regions of the triple helix. These include divalent crosslinks such as hydroxylysine-keto-norleucine (HLKNL) and trivalent crosslinks like hydroxylysyl-pyridinoline (PYD). The overall crosslinking density and the ratio of different crosslink types varies across species, tissues types and age, and is further altered by disease (Snedeker and Gautieri 2014). ColBuilder enables precise control over the composition and density of these crosslinks, accommodating various types and combinations (see Supplementary Fig. S5 for a full list of available structures).

The tool offers two specification methods. First, users can mix triple helices with different crosslink types by defining their ratios. The algorithm then randomly combines these helices to generate a microfibril with the desired composition. Second, ColBuilder can randomly remove existing crosslinks, replacing them with lysine residues using Chimera’s *swapaa* command (Pettersen *et al.* 2004), at a user-defined rate.

**Figure 3.**
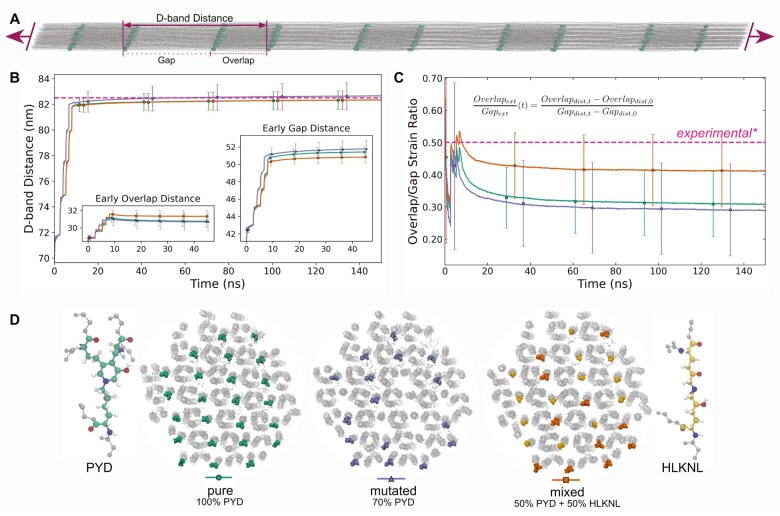
Validation of collagen models with molecular dynamics simulations. (A) Simulation snapshot of a 335-nm long pulled collagen fibril, illustrating multiple gap and overlap regions with crosslink atoms highlighted as green spheres. (B) D-band distance in nanometer and (C) overlap/gap strain ratio evolution with simulation time (ns) for different collagen models illustrated in (D) [insets in (B) show overlap and gap distances early in the simulation, when the fibril was going through greater structural change due to pulling]. (D) Cross-sections of collagen fibrils with pure (100% PYD), mutated (70% PYD), and mixed (50% PYD + 50% HLKNL) crosslinks. In both (B) and (C), experimentally reported values (Peacock and Kreplak 2019) are shown by the dashed pink line. Error bars represent the standard error of the mean, combining frame-wise variability and trajectory uncertainty, and are horizontally offset for clarity. Error magnitude remains approximately constant throughout the simulation, and line plots show true data. Detailed methods for error calculations are provided in the Supplementary Material.

Figure 3D shows three different crosslink setups generated based on the Bravais lattice: pure trivalent, trivalent with randomly removed crosslinks, and mixed divalent–trivalent. Trivalent PYD crosslinks are located at the transition between gap and overlap regions (Fig. 3A), linking adjacent triple helices. The cross-section of this microfibril reveals equally spaced crosslinks forming a rounded shape. The second setup demonstrates the capability of ColBuilder to generate models with varying crosslinking density. We reduced the number of PYD crosslinks within the pure trivalent crosslinked microfibril by randomly replacing 30% of the trivalent crosslinks with lysine residues. This resulted in a microfibril with 70% pure PYD crosslinks, featuring a significantly lower crosslink density compared to the other two microfibrils. For the mixed divalent–trivalent crosslinked microfibril, we defined four types of collagen molecules based on their crosslinks at each telopeptide region: divalent–divalent, trivalent–divalent, divalent–trivalent, and trivalent–trivalent. These were combined in equal proportions, resulting in a microfibril containing both divalent HLKNL and trivalent PYD crosslinks.

These approaches allow for fine-tuning of crosslink density and composition, which are expected to significantly affect the microfibril’s mechanical properties at both macro and microscopic scales (Kwansa *et al.* 2016, Rennekamp *et al.* 2023). Additionally, users can add their own crosslink structures to ColBuilder’s database, enabling them to model and study the impact of crosslinking variations on fibril structure and mechanics, critical for understanding conditions such as aging and diseases where crosslink patterns and types are altered (Monnier *et al.* 1996, Snedeker and Gautieri 2014).

## 3 Results

### 3.1 All-atom simulations validate models from ColBuilder

To validate the stability and force-extension behavior of the collagen microfibrils generated by ColBuilder, we performed all-atom MD simulations. To demonstrate the capabilities of ColBuilder, we chose a fibril length of 335 nm which covers the length of a full triple helix, resulting in five complete gap and four complete overlap regions. This goes far beyond previous all-atom MD simulations of one gap and one overlap region, the only system size available in our previous database (Obarska-Kosinska *et al.* 2021). We generated the microfibril topology using GROMACS 2023 (Van Der Spoel *et al.* 2005), combining the Amber99sb*-ildnp force field (Best and Hummer 2009, Lindorff-Larsen *et al.* 2010) for the protein and TIP3P for water with GROMACS’ *pdb2gmx* tool. The simulations of our system of approximately 43 million atoms were conducted using the GENESIS MD engine on the Fugaku supercomputer (Jung *et al.* 2021, 2024), achieving a performance of approximately 11 ns/day, which demonstrates the feasibility of simulating these large-scale biological systems.

Our protocol began with energy minimization in vacuum, followed by solvation and further minimization. We then equilibrated the system through a series of steps: (i) simulation in the canonical ensemble (NVT; number of particles (N), volume (V), and temperature (T) are constant) at 300 K over 10 ns using the stochastic velocity rescaling (SVR) (Bussi and Parrinello 2007) with gradually increasing time steps (0.5–2 fs) and (ii) 5 ns NPT (constant-temperature, constant-pressure ensemble) simulation at 1 atm and 300 K using the SVR thermostat and the Martyna–Tobias–Klein barostat (Martyna *et al.* 1994), with gradually increasing time steps (0.5–2 fs). Throughout equilibration, backbone atoms were position-restrained (force constant is decreased gradually from 2.5 to 1.0 kcal mol${}^{-1}$ Å${}^{-2}$ in the NVT equilibration and from 1.0 to 0.3 kcal mol${}^{-1}$ Å${}^{-2}$ in the NPT simulation) to maintain microfibril structure while allowing side-chain relaxation. Periodic boundary conditions were applied in all directions. We used group-based thermostat/barostat with optimal temperature evaluation (Jung *et al.* 2019, Jung and Sugita 2020).

To allow the 300 nm system to structurally adopt to the applied external force, we implemented a multistep constant force protocol, incrementally increasing force to 1 nN per strand over 16.5 ns, followed by a 495 ns sustained load simulation. This force magnitude was chosen based on estimated physiological loads on individual collagen molecules, corresponding to stresses in the tens of MP range (Komi 1990), and the duration was determined to be sufficient for observing initial structural reorganization based on previous studies (Zapp *et al.* 2020).

Figure 3A shows a snapshot of the collagen microfibril with trivalent PYD crosslinks under the applied force. Notably, no collagen molecule was pulled out during the simulation, demonstrating the structural integrity of our model. The snapshot also reveals differences in molecular packing and stretching between gap and overlap regions under force, with the overlap region showing more parallel alignment of collagen triple helices and less elongation compared to the more intertwined gap region.

We analyzed the mechanical response by measuring three key parameters: end-to-end distance of the entire microfibril, D-band distance—defined as the sum of overlap and gap region lengths—, and the overlap/gap strain ratio—calculated as the ratio between the extensions of overlap and gap regions. These measurements were conducted for three distinct crosslink configurations: pure trivalent, partly trivalent (70% trivalent, 30% absent), and mixed divalent–trivalent crosslinked microfibrils. This approach allowed comparison of their mechanical behaviors under load. To ensure reproducibility and assess variability, we performed three independent simulations for each crosslink configuration.

Our results show that all distance measures increased sharply at the onset of force application before asymptotically approaching constant values. The 335-nm long microfibril extended by approximately 23%, with the partly trivalent crosslinked microfibril stretching the most (>409 nm), followed closely by the pure trivalent and mixed crosslinked microfibrils (≈408 nm) (Supplementary Fig. S4).

The D-band stretching, resulting from the sum of gap and overlap region elongations, showed similar trends (Fig. 3B). We observed elongations of 82.4 and 82.8 nm for the pure trivalent/mixed divalent–trivalent and the partly trivalent crosslinked microfibrils, respectively. Notably, this 22% applied strain D-band elongation aligns well with both smaller scale AA simulations under force (82.5±1.0 nm) and atomic force microscopy (AFM) nanoindentation experiments from the literature (80–82.5 nm) (Minary-Jolandan and Yu 2009, Peacock and Kreplak 2019, Gachon and Mesquida 2020, Rennekamp *et al.* 2023).

The overlap region exhibited a unique stretching pattern (inset of Fig. 3B), with an initial sudden increase from 27.9 to 31.3 nm, followed by a gradual decrease to constant values of 30.3 and 30.7 nm for the mixed divalent–trivalent and the pure/partly trivalent crosslinked microfibrils, respectively. Concurrently, the gap region (also shown in the inset of Fig. 3B) expanded from 39.7 to 52.1 nm for the partly trivalent and mixed crosslinked microfibrils, and to 51.7 nm for the pure trivalent microfibril. These measurements yielded an average overlap-gap strain ratio of 25%–30% (Fig. 3C), suggesting that the overlap region is approximately three to four times stiffer than the gap region. This finding agrees well with AFM nanoindentation experiments reporting a 25%–100% higher stiffness in the overlap region compared to the gap region (Minary-Jolandan and Yu 2009, Peacock and Kreplak 2019).

It is important to note that these mechanical properties can vary depending on factors such as collagen type and tissue of origin (e.g. Achilles or rat tail tendon), experimental conditions (fibril humidity, temperature), and applied force or strain. Despite these potential variations, our collagen microfibrillar structure largely reproduces key structural and mechanical observables reported in the literature, particularly the D-band lengthening and the overlap-gap strain ratio. These results validate the structural integrity of the collagen microfibrils generated by ColBuilder, demonstrating their suitability for further studies of collagen mechanics and function under physiologically relevant conditions.

### 3.2 Computational performance

To assess the efficiency of ColBuilder, we analyzed its computational performance in relation to the size and complexity of the generated collagen microfibrils. We measured the wall clock time—the actual time taken to complete a run—for various configurations, focusing on how it scales with the number of collagen chains in the microfibril.

Our analysis revealed a strong correlation between the number of chains in the microfibril and the computational time required. This number of chains is directly influenced by both the contact distance parameter and the desired fibril length, making it a useful metric for predicting performance across different configurations.

Supplementary Figure S5 illustrates the relationship between the number of collagen chains the wall clock time and the contact distance (dc). We observed that the computational time increases exponentially with the number of chains/contact distance. To provide context for these numbers, we can relate them back to the physical parameters of the microfibril. A typical configuration with a radius of 12 nm (dc=45 Å) and a fibril length of 330 nm resulted in 271 chains and took around 6 min 34 s to generate. Increasing the contact distance to the double value (dc=90 Å), corresponding to a radius of 16.5 nm while maintaining the same length increased the chain count to 739 and the computational time to 51 min and 25 s.

These performance metrics were obtained using a standard desktop computer with an Intel Core i5-9600K processor, 3 × 8 GB of DIMM DDR4 RAM, and an NVIDIA Corporation TU106 GPU, running Ubuntu 22.04.4 LTS. Overall, this desktop computer cannot handle contact distance exceeding 100 Å or radii larger than 17 nm due to memory issues. It is worth noting that runtime may vary depending on the specific hardware configuration used. The observed computational efficiency of ColBuilder makes it a practical tool for researchers, enabling the efficient generation and iteration of collagen microfibril models for various studies in biomechanics and structural biology.

## 4 ColBuilder web server

In addition to the command line application, we provide a web server application of ColBuilder. The web server is implemented within the Flask framework and offers the full functionality of the command line version for selected species and crosslink combinations. ColBuilder web server is freely available at: https://colbuilder.mpip-mainz.mpg.de.

## 5 Conclusion

We have presented ColBuilder, a computational tool that automates the generation of collagen fibril structures with unprecedented control over their composition and architecture. The integration of homology modeling, higher order structure generation, and Bravais lattice optimization allows researchers to systematically explore sequence variations, crosslinking patterns, and dimensional parameters that were previously challenging to investigate. The tool’s web interface and command-line application make these capabilities accessible to both structural biologists and biophysics researchers, while its modular design facilitates future extensions.

The ColBuilder project is actively developing. Current work focuses on expanding the crosslink library to include advanced glycation end-product crosslinks and implementing topology generation for coarse-grained (Martini) force fields. The demonstrated capability to generate and simulate full-length fibrils establishes a foundation for investigating different collagen types and disease-specific modifications.

The current starting point of ColBuilder is a model from X-ray scattering (Orgel *et al.* 2006). ColBuilder will greatly profit from additional knowledge on collagen’s hierarchical structure from other sources, including electron microscopy, new data on crosslinking or other posttranslational modifications from mass spectrometry, as well as complementary insights from structure prediction methods like AlphaFold (Jumper *et al.* 2021). While our preliminary analysis shows that current deep learning methods such as AlphaFold 3 struggle with collagen’s unique structural features, particularly at interfaces where crosslinks form, targeted integration of these approaches could enhance future versions of ColBuilder. These developments will enable increasingly faithful computational studies of collagen mechanics across scales, from molecular interactions to tissue-level properties.

## Supplementary Material

btaf278_Supplementary_Data

## Data Availability

FASTA files for collagen Type I of all species discussed in this study are available in the ColBuilder GitHub repository. Scripts used in our simulations and code used for computational analyses are available in https://github.com/debora-monego/colbuilder-paper.

## References

[btaf278-B1] Abramson J , Adler J, Dunger J et al Accurate structure prediction of biomolecular interactions with Alphafold 3. Nature 2024;630:493–500. 38718835 10.1038/s41586-024-07487-w PMC11168924

[btaf278-B2] Avery NC , Sims TJ, Bailey AJ. Quantitative determination of collagen cross-links. In: Even-Ram, S., Artym, V. (eds), *Extracellular Matrix Protocols. Methods in Molecular Biology*, Vol 522. 2nd edn. Totowa, NJ: Humana Press, 2009, 103–21. 10.1007/978-1-59745-413-1_6 19247601

[btaf278-B3] Bella J , Eaton M, Brodsky B et al Crystal and molecular structure of a collagen-like peptide at 1.9 å resolution. Science 1994;266:75–81. 7695699 10.1126/science.7695699

[btaf278-B4] Best RB , Hummer G. Optimized molecular dynamics force fields applied to the helix- coil transition of polypeptides. J Phys Chem B 2009;113:9004–15. 19514729 10.1021/jp901540t PMC3115786

[btaf278-B5] Bussi G , Parrinello M. Accurate sampling using Langevin dynamics. Phys Rev E Stat Nonlin Soft Matter Phys 2007;75:056707. 17677198 10.1103/PhysRevE.75.056707

[btaf278-B6] Cock PJA , Antao T, Chang JT et al Biopython: freely available python tools for computational molecular biology and bioinformatics. Bioinformatics 2009;25:1422–3. 10.1093/bioinformatics/btp163 19304878 PMC2682512

[btaf278-B7] Depalle B , Qin Z, Shefelbine SJ et al Influence of cross-link structure, density and mechanical properties in the mesoscale deformation mechanisms of collagen fibrils. J Mech Behav Biomed Mater 2015;52:1–13. 10.1016/j.jmbbm.2014.07.008 25153614 PMC4653952

[btaf278-B8] Edgar RC. Muscle: multiple sequence alignment with high accuracy and high throughput. Nucleic Acids Res 2004;32:1792–7. 10.1093/nar/gkh340 15034147 PMC390337

[btaf278-B9] Eyre DR , Wu J-J. Collagen cross-links. In: Brinckmann J, Notbohm H, Müller PK (eds), *Collagen: Primer in Structure, Processing and Assembly. Topics in Current Chemistry*, Vol 247, 1st edn. Berlin Heidelberg: Springer, 2005, 207–29.

[btaf278-B10] Fratzl P , Fratzl-Zelman N, Klaushofer K. Collagen packing and mineralization. an X-ray scattering investigation of Turkey leg tendon. Biophys J 1993;64:260–6. 8431546 10.1016/S0006-3495(93)81362-6 PMC1262322

[btaf278-B11] Gachon E , Mesquida P. Stretching single collagen fibrils reveals nonlinear mechanical behavior. Biophys J 2020;118:1401–8. 32070478 10.1016/j.bpj.2020.01.038 PMC7091508

[btaf278-B12] Gautieri A , Vesentini S, Redaelli A et al Hierarchical structure and nanomechanics of collagen microfibrils from the atomistic scale up. Nano Lett 2011;11:757–66. 21207932 10.1021/nl103943u

[btaf278-B13] Jelinski LW , Sullivan C, Torchia D. 2h NMR study of molecular motion in collagen fibrils. Nature 1980;284:531–4. 7366722 10.1038/284531a0

[btaf278-B14] Jumper J , Evans R, Pritzel A et al Highly accurate protein structure prediction with Alphafold. Nature 2021;596:583–9. 34265844 10.1038/s41586-021-03819-2 PMC8371605

[btaf278-B15] Jung J , Sugita Y. Group-based evaluation of temperature and pressure for molecular dynamics simulation with a large time step. J Chem Phys 2020;153:234115. 33353318 10.1063/5.0027873

[btaf278-B16] Jung J , Kobayashi C, Sugita Y. Optimal temperature evaluation in molecular dynamics simulations with a large time step. J Chem Theory Comput 2019;15:84–94. 30468577 10.1021/acs.jctc.8b00874

[btaf278-B17] Jung J , Kobayashi C, Kasahara K et al New parallel computing algorithm of molecular dynamics for extremely huge scale biological systems. J. Comput. Chem 2021;42:231–41. 33200457 10.1002/jcc.26450 PMC7975918

[btaf278-B18] Jung J , Yagi K, Tan C et al Genesis 2.1: high-performance molecular dynamics software for enhanced sampling and free-energy calculations for atomistic, coarse-grained, and quantum mechanics/molecular mechanics models. J. Phys. Chem. B 2024;128:6028–48. 38876465 10.1021/acs.jpcb.4c02096 PMC11215777

[btaf278-B19] Kadler KE , Holmes DF, Trotter JA et al Collagen fibril formation. Biochem J 1996;316:1–11. 8645190 10.1042/bj3160001 PMC1217307

[btaf278-B20] Komi PV. Relevance of in vivo force measurements to human biomechanics. J Biomech 1990;23:23–34. 2081741 10.1016/0021-9290(90)90038-5

[btaf278-B21] Kwansa AL , De Vita R, Freeman JW. Tensile mechanical properties of collagen type I and its enzymatic crosslinks. Biophys Chem 2016;214-215:1–10. 27160969 10.1016/j.bpc.2016.04.001

[btaf278-B22] Lee J , Patel DS, Ståhle J et al CHARMM-GUI membrane builder for complex biological membrane simulations with glycolipids and lipoglycans. J. Chem. Theory Comput 2019;15:775–86. 30525595 10.1021/acs.jctc.8b01066

[btaf278-B23] Lindorff-Larsen K , Piana S, Palmo K et al Improved side-chain torsion potentials for the Amber ff99SB protein force field. Proteins 2010;78:1950–8. 20408171 10.1002/prot.22711 PMC2970904

[btaf278-B24] Macqueen J. Some methods for classification and analysis of multivariate observations. In: *Proceedings of 5th Berkeley Symposium on Mathematical Statistics and Probability*. University of California Press, 1967, 281–97.

[btaf278-B25] Martínez L , Andrade R, Birgin EG et al Packmol: a package for building initial configurations for molecular dynamics simulations. J Comput Chem 2009;30:2157–64. 19229944 10.1002/jcc.21224

[btaf278-B26] Martyna GJ , Tobias DJ, Klein ML. Constant pressure molecular dynamics algorithms. J. Chem. Phys 1994;101:4177–89.

[btaf278-B27] Minary-Jolandan M , Yu M-F. Nanomechanical heterogeneity in the gap and overlap regions of type I collagen fibrils with implications for bone heterogeneity. Biomacromolecules 2009;10:2565–70. 19694448 10.1021/bm900519v

[btaf278-B28] Monnier VM , Glomb M, Elgawish A et al The mechanism of collagen cross-linking in diabetes: a puzzle nearing resolution. Diabetes 1996;45:S67–72. 8674897 10.2337/diab.45.3.s67

[btaf278-B29] Monti S , Bronco S, Cappelli C. Toward the supramolecular structure of collagen: a molecular dynamics approach. J Phys Chem B 2005;109:11389–98. 16852392 10.1021/jp0440941

[btaf278-B30] Obarska-Kosinska A , Rennekamp B, Ünal A et al ColBuilder: a server to build collagen fibril models. Biophys J 2021;120:3544–9. 34265261 10.1016/j.bpj.2021.07.009 PMC8456305

[btaf278-B31] Orgel JP , Irving TC, Miller A et al Microfibrillar structure of type I collagen in situ. Proc Natl Acad Sci U S A 2006;103:9001–5. 16751282 10.1073/pnas.0502718103 PMC1473175

[btaf278-B32] Peacock CJ , Kreplak L. Nanomechanical mapping of single collagen fibrils under tension. Nanoscale 2019;11:14417–25. 31334733 10.1039/c9nr02644d

[btaf278-B33] Pedregosa F , Varoquaux G, Gramfort A et al Scikit-learn: machine learning in Python. J Mach Learn Res 2011;12:2825–30.

[btaf278-B34] Peixoto PDS , Laurent G, Azaïs T et al Solid-state NMR study reveals collagen I structural modifications of amino acid side chains upon fibrillogenesis. J Biol Chem 2013;288:7528–35. 23341452 10.1074/jbc.M112.390146 PMC3597793

[btaf278-B35] Perumal S , Antipova O, Orgel JP. Collagen fibril architecture, domain organization, and triple-helical conformation govern its proteolysis. Proc Natl Acad Sci U S A 2008;105:2824–9. 18287018 10.1073/pnas.0710588105 PMC2268544

[btaf278-B36] Pettersen EF , Goddard TD, Huang CC et al UCSF chimera—a visualization system for exploratory research and analysis. J Comput Chem 2004;25:1605–12. 10.1002/jcc.20084 15264254

[btaf278-B37] Qi Y , Lee J, Klauda JB et al CHARMM-GUI nanodisc builder for modeling and simulation of various nanodisc systems. J Comput Chem 2019;40:893–9. 30677169 10.1002/jcc.25773

[btaf278-B38] Rennekamp B , Karfusehr C, Kurth M et al Collagen breaks at weak sacrificial bonds taming its mechanoradicals. Nat Commun 2023;14:2075. 37045839 10.1038/s41467-023-37726-z PMC10097693

[btaf278-B39] Shoulders MD , Raines RT. Collagen structure and stability. Annu Rev Biochem 2009;78:929–58. 19344236 10.1146/annurev.biochem.77.032207.120833 PMC2846778

[btaf278-B40] Snedeker JG , Gautieri A. The role of collagen crosslinks in ageing and diabetes—the good, the bad, and the ugly. Muscles Ligaments Tendons J 2014;4:303–8. 25489547 PMC4241420

[btaf278-B41] Streeter I , de Leeuw NH. Atomistic modeling of collagen proteins in their fibrillar environment. J Phys Chem B 2010;114:13263–70. 20873729 10.1021/jp1059984 PMC3505825

[btaf278-B42] Streeter I , de Leeuw NH. A molecular dynamics study of the interprotein interactions in collagen fibrils. Soft Matter 2011;7:3373–82. 10.1039/C0SM01192D 23526918 PMC3605786

[btaf278-B43] Uzel SG , Buehler MJ. Molecular structure, mechanical behavior and failure mechanism of the c-terminal cross-link domain in type I collagen. J Mech Behav Biomed Mater 2011;4:153–61. 21262493 10.1016/j.jmbbm.2010.07.003

[btaf278-B44] Van Der Spoel D , Lindahl E, Hess B et al Gromacs: fast, flexible, and free. J Comput Chem 2005;26:1701–18. 16211538 10.1002/jcc.20291

[btaf278-B45] Willett TL , Labow RS, Aldous IG et al Changes in collagen with aging maintain molecular stability after overload: evidence from an in vitro tendon model. 2010;132:031002. 10.1115/1.4000933 20459190

[btaf278-B46] Zapp C , Obarska-Kosinska A, Rennekamp B et al Mechanoradicals in tensed tendon collagen as a source of oxidative stress. Nat. Commun 2020;11:2315. 32385229 10.1038/s41467-020-15567-4 PMC7210969

