# Supplementary material for: ColBuilder: flexible structure generation of crosslinked collagen fibrils
Source: Bioinformatics. 2025 May 5;41(6):btaf278. doi: 10.1093/bioinformatics/btaf278 (PMC12148304; doi:10.1093/bioinformatics/btaf278)
Supplement: btaf278_Supplementary_Data [file btaf278_supplementary_data.zip › ColBuilder - SupplementaryInformation.pdf]

ColBuilder: Flexible structure generation of crosslinked collagen fibrils

Debora Monego[1,2,*,†], Matthias Brosz[2,3,†], Johanna Buck[1,2,3], Vsevolod Viliuga[1,5,6], Paulius Greicius [1,4], Jaewoon Jung[7,8], Torsten Stuehn[1], Matthias Schmies[1], Yuji Sugita[7,8], Frauke Gräter[1,2,3,*]

1 Max Planck Institute for Polymer Research, Ackermannweg 10, Mainz, Germany
2 Heidelberg Institute for Theoretical Studies, Am Schloss-Wolfsbrunnenweg 35, Heidelberg, Germany
3 Institute for Scientific Computing, Heidelberg University, Im Neuenheimer Feld 205, Heidelberg, Germany
4 Max Planck School Matter to Life, Jahnstrasse 29, 69120, Heidelberg, Germany
5 Science for Life Laboratory, Tomtebodavägen 23, Solna, Sweden
6 Department of Biochemistry and Biophysics, Stockholm University, Svante Arrhenius Väg 16C, Stockholm, Sweden
7 Computational Biophysics Research Team, RIKEN Center for Computational Science, Kobe, Yogo, Japan
8 Theoretical Molecular Science Laboratory, RIKEN Center for Computational Science, Wako, Saitama, Japan

† These authors contributed equally to this work
*** Corresponding authors: frauke.graeter@mpip-mainz.mpg.de, monegod@mpip-mainz.mpg.de**

Homology Modeling and Initial Structure Generation

Collagen triple helices for various species were modeled using ColBuilder, with *Rattus novergicus* collagen type I as the reference. Sequence alignment was performed to assess conservation, and per-residue RMSD values were calculated to quantify structural differences. As shown in Figure S1, the RMSD values for the human and rat collagen chains remain below 1.5 Å, indicating a high level of structural accuracy in our homology models.

To further evaluate the consistency of the models across species, Table S1 shows the average sequence identity, per-residue RMSD, and axial rise per triplet for collagen triple helices generated for all species in our database. These values demonstrate consistent homology model quality, with RMSD values typically ranging between 1.0-1.4 Å, supporting the reliability of our homology modeling procedure across a diverse set of species.

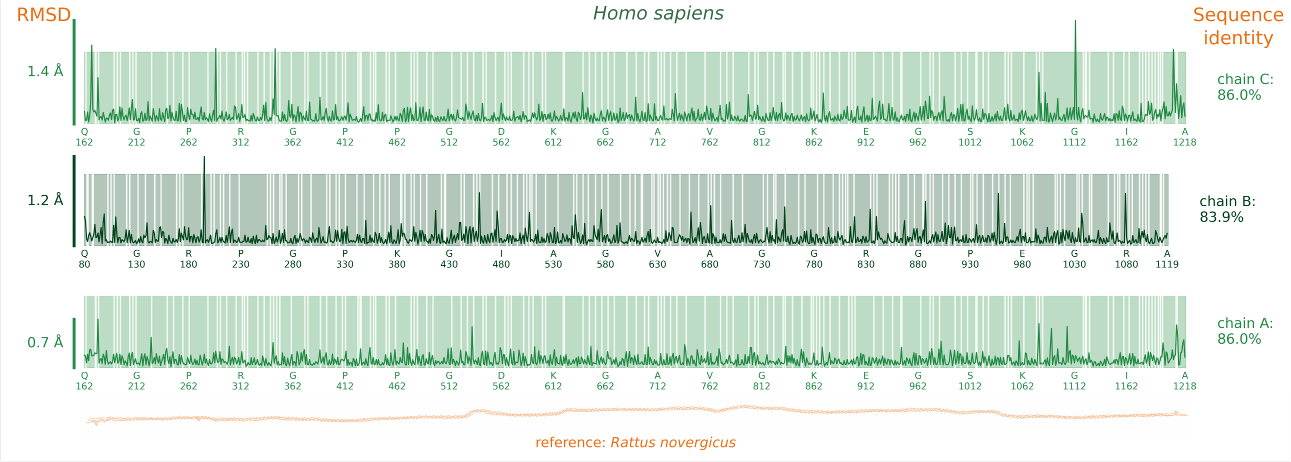

Figure S1. Sequence alignment (solid color: identical, and gaps: diverging residues) and per-residue RMSD (line) between each chain of human (*Homo sapiens*) and rat (*Rattus novergicus*) collagen type I, shown at the bottom for reference. The magnitude of the RMSD (Å) is shown on the left, and the average sequence identity for each chain on the right.

Table S1. Average sequence identity, per-residue RMSD ± SD, and average axial rise per triplet ± SD for collagen triple helices generated with ColBuilder. Collagen type I of *Rattus novergicus* was used as a reference.

Species	Sequence Identity, %	RMSD, Å	Axial Rise per Triplet, Å
Otolemur garnettii	93.57	1.25 ± 0.32	9.29 ± 0.46
Xiphophorus maculatus	72.01	1.39 ± 0.10	9.26 ± 0.45
Tetraodonni groviridis	71.6	1.35 ± 0.09	9.24 ± 0.37
Oreochromis niloticus	75.04	1.29 ± 0.08	9.28 ± 0.45
Callithrix jacchus	94.81	1.22 ± 0.3	9.27 ± 0.44
Pongo abelii	93.55	1.25 ± 0.17	9.28 ± 0.45
Pelodiscus sinensis	83.29	1.24 ± 0.16	9.23 ± 0.38
Pan troglodytes	94.72	1.08 ± 0.28	9.28 ± 0.44
Mustela putorius	95.32	1.06 ± 0.29	9.27 ± 0.44
Canis lupus	95.29	1.08 ± 0.28	9.28 ± 0.44
Ailuropoda melanoleuca	93.74	1.28 ± 0.13	9.27 ± 0.50
Homo sapiens	85.3	1.12 ± 0.32	9.28 ± 0.44
Danio rerio	75.43	1.23 ± 0.01	9.28 ± 0.46
Mus musculus	97.64	1.12 ± 0.26	9.29 ± 0.46
Loxodonta africana	90.17	1.22 ± 0.20	9.25 ± 0.43
Bos taurus	94.49	1.08 ± 0.27	9.3 ± 0.44
Oryzias latipes	75.04	1.29 ± 0.08	9.28 ± 0.45
Myotis lucifugus	94.5	1.08 ± 0.28	9.29 ± 0.46

Evaluation of AlphaFold 3 for Collagen Structure Prediction

We evaluated AlphaFold 3's [1] ability to predict collagen structures by focusing on five key regions from collagen type I sequences of three species: *Rattus norvegicus*, *Homo sapiens*, and *Tetraodon nigroviridis*. These species were selected to represent varying evolutionary distances while including both mammals and non-mammals.

The following regions were submitted to AlphaFold 3:

- **H**: Central helical region (residues 495-545)
- **NT**: N-terminal region (residues 0-50)
- **NTX**: N-terminal crosslinking interface (combination of N-terminal regions of one triple helix and C-terminal regions of another)
- **CT**: C-terminal region (residues 1006-end for $\alpha 1$, 990-end for $\alpha 2$)
- **CTX**: C-terminal crosslinking interface (combination of C-terminal regions of one triple helix and N-terminal regions of another)

We report both pTM (predicted Template Modeling) and ipTM (interface predicted Template Modeling) scores for all predictions. While pTM measures the overall structural confidence of the entire model, ipTM specifically evaluates the confidence of predicted interactions between chains at protein interfaces. For collagen, which derives its stability from interchain interactions in the triple helix and between adjacent molecules, the ipTM score is particularly relevant for assessing the quality of predictions at these critical interfaces.

As shown in Table S2, AlphaFold 3 predicted individual collagen regions (NT, CT, H) with moderate confidence (pTM scores of 0.67-0.75). However, prediction quality decreased substantially for crosslinking interfaces (NTX, CTX), with pTM scores ranging from 0.45-0.51 for N-terminal and 0.45-0.49 for C-terminal crosslinking interfaces. According to AlphaFold documentation, pTM scores below 0.5 or ipTM values below 0.6 suggest a failed prediction, indicating that the crosslinking interface predictions fall into this low-confidence category.

The decreased prediction quality at interfaces is particularly evident when examining the predicted structures (Figure S2). In these models, lysine residues that should facilitate crosslinks between adjacent collagen molecules are positioned too far apart for crosslink formation. This failure to correctly model the interfacial regions is critical, as proper crosslinking is essential for fibril stability and mechanical properties of collagen matrices.

Overall, prediction quality showed minimal variation across species, with human and rat collagen sequences yielding slightly higher confidence predictions than fish (*Tetraodon*) for individual regions. However, all species exhibited similar

Table S2. AlphaFold 3 prediction confidence metrics for collagen structures from three species.

Organism	Region	ipTM	pTM
Homo sapiens	H	0.67	0.70
	NT	0.71	0.74
	NTX	0.40	0.46
	CT	0.66	0.68
	CTX	0.39	0.45
Rattus norvegicus	H	0.69	0.71
	NT	0.73	0.75
	NTX	0.41	0.48
	CT	0.65	0.67
	CTX	0.44	0.49
Tetraodon nigroviridis	H	0.68	0.70
	NT	0.72	0.73
	NTX	0.45	0.51
	CT	0.70	0.71
	CTX	0.39	0.45

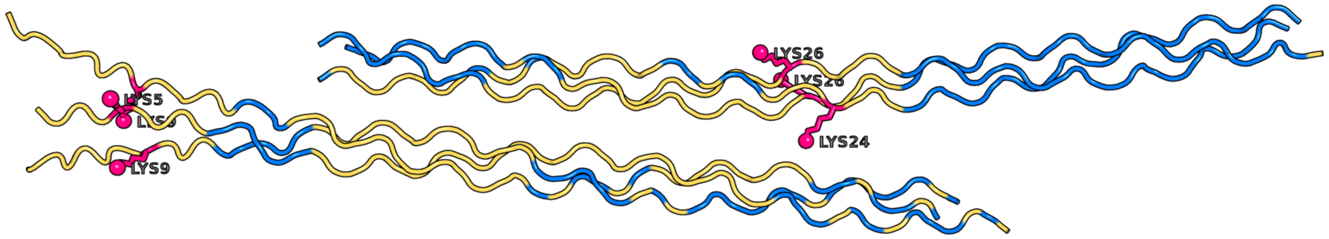

Figure S2. AlphaFold 3 prediction of a rat collagen structure (NTX) showing lower confidence prediction (plDDT score < 70, in yellow) for interfacial and telopeptide regions. Note the substantial distance between lysine residues (highlighted in magenta) that would need to be in proximity for crosslink formation.

patterns of decreased confidence at crosslinking interfaces, suggesting that these limitations in prediction are fundamental to collagen's complex quaternary structure rather than sequence-specific. These findings provide strong justification for our implementation of a template-based homology modeling approach in ColBuilder, which can explicitly model these critical crosslinking interfaces based on experimental structures.

Higher-Order Crystal Structural Generation

Contact distance parameter

The radius of collagen fibrils was determined using a geometric approach based on the spatial distribution of atoms in the PDB files. The x and y coordinates of all atoms were extracted to create a 2D projection of the fibril structure. A convex hull was then computed from these coordinates using SciPy's *ConvexHull* function, representing the outer boundary of the fibril cross-section. The centroid of all atomic coordinates was calculated as the mean of all x and y coordinates. Distances from this centroid to each point on the convex hull were computed, and the radius of the fibril was defined as the 95th percentile of these distances. This approach was chosen to provide a robust estimate of the fibril's outer boundary while accounting for potential outliers. To quantify the uncertainty in the radius calculation, we used the interquartile range (IQR) of the centroid-to-hull distances. The radius error was calculated as half of the IQR, offering a conservative estimate of the measurement uncertainty that is resistant to outliers. This method allows for a robust estimation of fibril radius that is less sensitive to local irregularities in the atomic coordinates, while providing a reliable measure of the variability in the fibril's

cross-sectional shape.

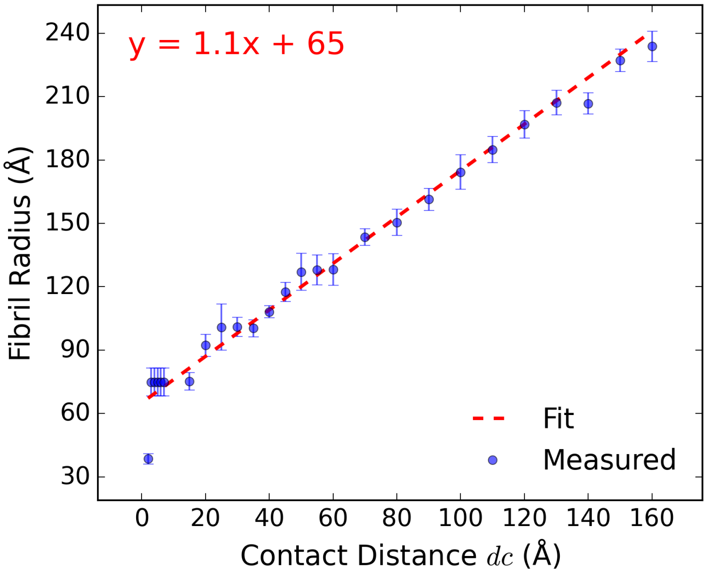

Figure S3. Dependence of fibril radius (Å) with ColBuilder.0 parameter contact distance *dc* (Å). Users can determine the approximate *dc* value needed to produce a fibril of a given radius by solving for x in the regression equation for the fitting.

All-atom simulations

The end-to-end distance was calculated as the Euclidean distance between the mean positions of the ACE (N-terminal) and NME (C-terminal) cap residues. This measure represents the overall extension of the collagen molecule.

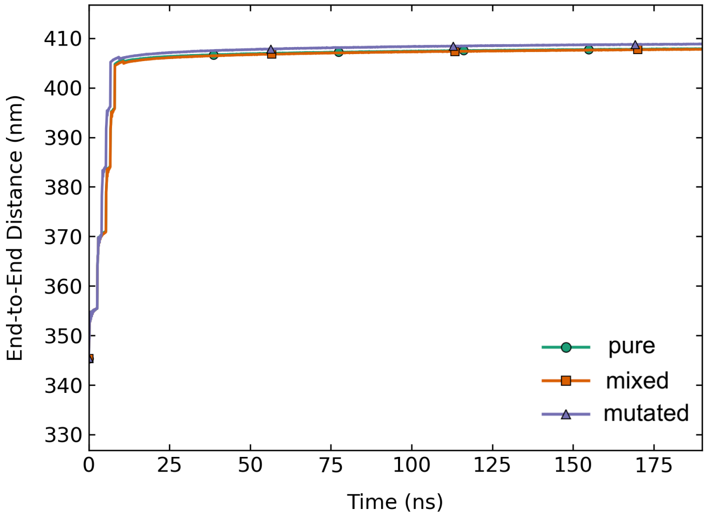

Figure S4. Overall extension of collagen fibril, represented by the end-to-end distance (nm) for pure (green, 100% PYD crosslinks), mutated (purple, 70% PYD) and mixed (orange, 50% PYD and 50% HLKNL) collagen fibrils. End-to-end distances were calculated as the Euclidian distance between the mean positions of the ACE (N-terminal) and NME (C-terminal) cap residues.

The error bars in Figure 3 B and C in the main text represent the standard error of the mean for D-band distances and overlap/gap strain ratios in our collagen molecular dynamics simulations. We used K-means clustering to identify cross-linking

residues and determine overlap and gap regions for each simulation frame. Standard errors for overlap and gap measurements were calculated from the spread of residue positions within clusters. For overlap/gap strain ratios, we propagated these errors using standard techniques. D-band distance errors, in turn, combine two components: (1) the propagated frame-wise overlap and gap errors, and (2) a bootstrap error estimate. The latter used a 10-frame sliding window with 1000 resamples per window. We combined these components using a root-mean-square method. This approach captures both local configurational variability and broader trajectory fluctuations, providing an integrated representation of uncertainty in our simulated collagen systems.

Crosslinks

ColBuilder uses a standardized format to define crosslinks between collagen molecules. Each crosslink is specified by a combination of its chemical type (Figure S5), the residues involved, and their precise connection points at the atomic level. The definition includes information about the original residue (typically lysine), its terminal location (N or C), the specific residue positions involved in the crosslink, and the detailed atomic connections. For trivalent crosslinks like PYD, three connection points are specified, while divalent crosslinks like HLKNL require only two. Table S3 shows examples of these definitions for *Homo sapiens*, illustrating the format for different crosslink types.

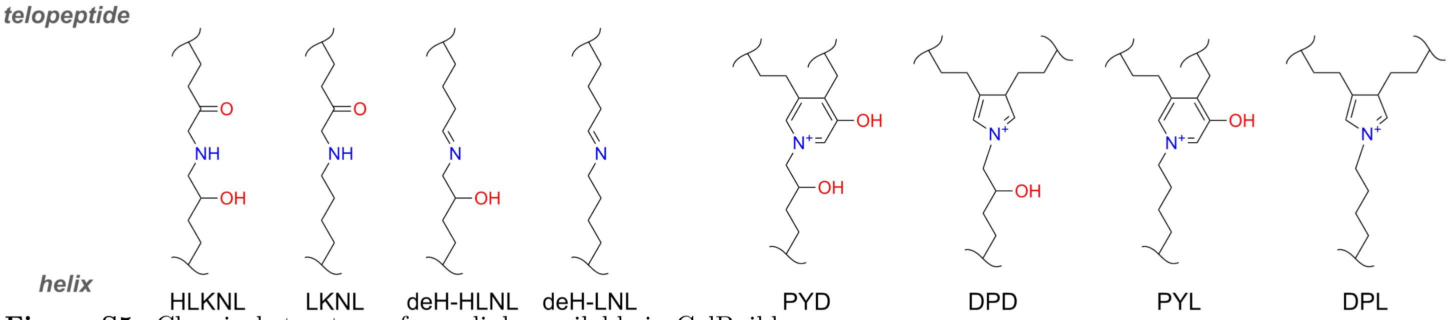

Figure S5. Chemical structure of crosslinks available in ColBuilder.

Table S3. Example of crosslink definitions in ColBuilder for *Homo sapiens*. Fields are: OR (original residue), terminal (N/C terminal), combination (residue positions involved), type (crosslink type), R1/A1/P1 (first residue/atom/position), R2/A2/P2 (second residue/atom/position), and R3/A31/A32/P3 (third residue/atoms/position for trivalent crosslinks).

OR	term	combination	type	R1	A1	P1	R2	A2	P2	R3	A31	A32	P3
LYS	C	1047.C-1047.A-104.C	PYD	LY3	CG	1047.C	LY2	CB	1047.A	LYX	C13	C12	104.C
LYS	N	9.C-5.B-944.B	PYD	LY3	CG	9.C	LY2	CB	5.B	LYX	C13	C12	944.B
LYS	C	1047.C-104.C	HLKNL	L5Y	NZ	1047.C	L4Y	CE	104.C	NONE	NONE	NONE	NONE
LYS	N	5.B-944.B	HLKNL	L5Y	NZ	5.B	L4Y	CE	944.B	NONE	NONE	NONE	NONE
LYS	C	1047.C-104.C	NOCROSS	NONE	NONE	NONE	NONE	NONE	NONE	NONE	NONE	NONE	NONE

Notes: PYD = pyridinoline (trivalent), HLKNL = hydroxylysinonorleucine (divalent), NOCROSS = no crosslink. Positions are given in residue.chain format. For trivalent crosslinks, R3/A31/A32/P3 specify the third connection point (A1 binds to A31 and A2 binds to A32, while divalent crosslinks use NONE for these fields.

Computational Performance

We evaluated ColBuilder's computational efficiency by measuring wall clock time as a function of system size, characterized by both contact distance (dc) and the resulting number of triple helices. As shown in Figure S6, the computational time increases exponentially with contact distance, reflecting the growing complexity of structure generation and optimization as more helices are included in the fibril.

System Specifications:

Processor: Intel Core i5-9600K processor, CPU family: 6, Model:158, Thread(s) per core: 1, Core(s) per socket: 6, Socket(s): 1, Stepping: 12, @ 3.70GHz

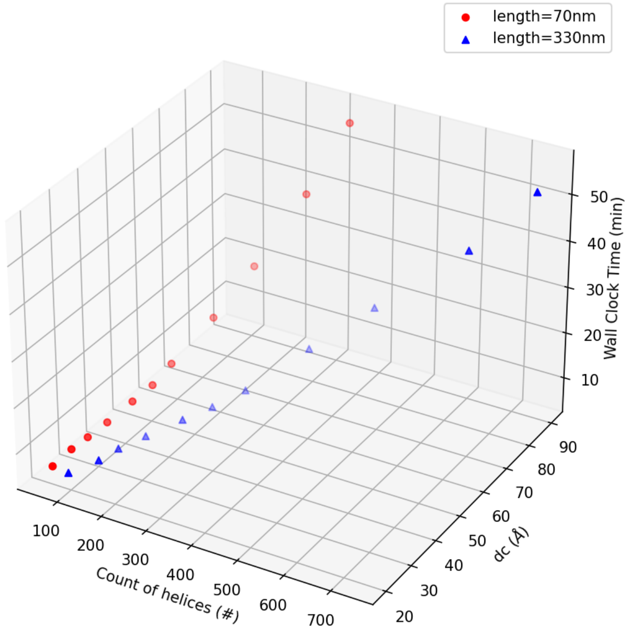

Figure S6. The wall clock time vs. the contact distance dc and count of helices. The figure shows the exponential behavior of the time vs. the contact distance for both shown lengths of fibrils.

RAM: 3x DIMM DDR4 Synchronous Unbuffered ,size: 8GB, width: 64 bits,clock: 2666MHz (0.4ns)
GPU: NVIDIA Corporation TU106 GPU (NVIDIA-SMI 550.107.02), running Ubuntu 22.04.4 LTS

References

1. Josh Abramson, Jonas Adler, Jack Dunger, Richard Evans, Tim Green, Alexander Pritzel, Olaf Ronneberger, Lindsay Willmore, Andrew J Ballard, Joshua Bambrick, et al. Accurate structure prediction of biomolecular interactions with alphafold 3. *Nature*, 630:493–500, 2024.
